# Editorial: Recent Advances of Near Infrared Applications in Fruits and Byproducts

**DOI:** 10.3389/fpls.2022.858040

**Published:** 2022-02-28

**Authors:** José Miguel Hernández-Hierro, Daniel Cozzolino, Chao-Hui Feng, Ana Elisa Rato, Julio Nogales-Bueno

**Affiliations:** ^1^Food Colour and Quality Laboratory, Department of Nutrition and Food Science, Facultad de Farmacia, Universidad de Sevilla, Seville, Spain; ^2^Centre for Nutrition and Food Sciences, Queensland Alliance for Agriculture and Food Innovation, The University of Queensland, Saint Lucia, QLD, Australia; ^3^School of Regional Innovation and Social Design Engineering, Faculty of Engineering, Kitami Institute of Technology, Kitami, Japan; ^4^RIKEN Centre for Advanced Photonics, RIKEN, Sendai, Japan; ^5^MED – Mediterranean Institute for Agriculture, Environment and Development & Departamento de Fitotecnia, Escola de Ciências e Tecnologia, Universidade de Évora, Evora, Portugal; ^6^Food Colour and Quality Laboratory, Department of Analytical Chemistry, Facultad de Farmacia, Universidad de Sevilla, Seville, Spain

**Keywords:** near infrared, spectroscopy, hyperspectral imaging, chemometrics, fruits, vegetables

Near-infrared spectroscopy (NIRS) has been widely applied to fruit, crops, and vegetable-products to ensure their safety and quality. In the last decades, mainly benchtop devices have been utilized for the development of *at-line* methods for qualitative and quantitative control. These methods have been limited by the chemical and physical heterogeneity of samples with high moisture content and high seasonal and regional variations. Recent advances in NIRS, such as improvements in sensors, new grating techniques, new chemometric procedures, etc, have allowed the emergence of new devices and methodologies. Simpler, faster, and more accurate models are being obtained. In addition, more complex parameters are being tested (detection of contaminants, extractability of interesting compounds, or characterization of individual compounds within a family). Infrared spectroscopy is therefore continually being renewed and provides new methods and tools for the fruit and vegetable industry.

This topic focuses on the recent advances applied for quality control in fruits and vegetables. These advances can be directly related to the spectral device, to the chemometric approach applied, to the application developed, or to a combination of all of them.

Two studies are dedicated to quality control of berries, namely strawberry and table grape. Su et al. collected visible and near-infrared spectra of strawberries with a hyperspectral device. This imaging system coupled with deep learning was used to identify the maturity degree of strawberries and estimate the soluble solid contents (SSC) of the fruit. They applied a convolutional neural network (CNN) architecture named Residual Network (ResNet). One-dimension residual neural network (1D ResNet) and three-dimension (3D) ResNet were built using 1D spectra and 3D hyperspectral image as inputs for maturity degree evaluation. Good performances were obtained for maturity degree identification, with the classification accuracy over 90% in the training set and classification accuracy over 84% in the validation and testing sets for both 1D and 3D ResNet. For classification, 1D and 3D ResNet showed close results, however, the computation cost of 3D ResNet was higher than that of 1D ResNet. The results indicated that 1D spectra were able to identify strawberry maturity degree and determine SSC with a small number of samples. Daniels et al. used a MATRIX-F Fourier Transform FT-NIR spectrometer (800–2,500 nm) to acquire the spectral information of table grape bunches. Their objective was to detect the presence or absence of different browning phenotypes in grape samples with different periods of cold storage. To achieve that goal, they applied a novel chemometric approach based on analysis of neural networks (ANN) and partial least squares discriminant analysis (PLS-DA). They use this combination of spectral and chemometric tools to study two variations in the phenotypic manifestation of grape berry browning: friction browning, i.e., a form of external browning that table grape berries develop as a result of rolling against each other, and chocolate browning, a form of internal browning that entails a discoloration mostly at the stylar-end of the berry. They both represent an interesting challenge since currently they can only be visually detected through vigorous inspection of the bunches. NIR spectroscopy, coupled with PLS-DA analysis and the machine learning technique ANN, showed that chocolate browning classification is more accurate than friction browning in table grapes.

Arendse et al. applied NIR spectroscopy to pomegranate juice. This fruit juice is a rich source of bioactive compounds and has received considerable interest in research and consumption during the last 15 years. The extraction method of this juice has a strong influence on its nutritive composition and bioactive properties. In this study, attenuated total reflectance Fourier transform NIR (ATR-FT-NIR) spectroscopy was used to classify pomegranate juice extracted through different methods. To do that, orthogonal partial least squares discriminant analysis (OPLS-DA) was applied and a classification accuracy of 100% was achieved between the different extraction methods studied. Therefore, the developed method using ATR-FT-NIR spectroscopy can be a good starting point for the development of methods for online and inline grading of pomegranate juice.

Another important group of fruits is the nuts group. This topic contains a study using NIR hyperspectral imaging for quality control of walnuts. Nogales-Bueno et al. used this tool to control the fatty acid profile in walnuts. They applied linear discriminant analysis (LDA) to extract walnut spectra from the spectral images, principal component analysis (PCA) to sort the spectral information and detect spectral outliers, and partial least square (PLS) regression to model the relationship between the spectral and fatty acid profiles. They modeled total fat, saturated, monounsaturated, polyunsaturated, and individual fatty acids. As a result, NIR hyperspectral imaging has shown to be a promising tool for the screening of total fat, saturated, monounsaturated, and polyunsaturated fatty acids and even for some individual fatty acids such as palmitic, margaric, oleic, linolelaidic, linoleic, and α-linolenic acids.

Finally, a study where the main spectral tool applied was Fourier transform mid-infrared (FT-MIR) spectroscopy is also included in this Research Topic. The MIR region contains the fundamental vibrations that produce overtones and combination bands typical of the NIR region. The two regions are therefore closely related. Liu et al. collected the MIR spectra of wild medicinal plants (*Gentiana rigescens*) with the aim of controlling the geographical origin and content of secoiridoids of these samples. For that, they applied PLS-DA to identify the sample origin and PLS regression for the quantitative control of the secoiridoids content. Different spectral pre-treatments were tested. In addition, an emerging pre-processing fusion approach, known as sequential pre-processing through orthogonalization (SPORT), was applied. SPORT was carried out to integrate the complementary information linked to different pre-processing techniques for the qualitative and quantitative assessment of wild medicinal plants. The application of the SPORT approach gave much better results than those obtained with traditionally pre-treated spectra.

Several interesting applications have been described and tested in this Research Topic. In these applications, infrared spectroscopy has made it possible to monitor the quality of different fruits and vegetables from a quantitative or qualitative point of view. However, further work is needed to move toward more robust and reliable non-destructive methods.

## Author Contributions

JN-B wrote and revised the manuscript. JH-H carefully revised and edited the manuscript. DC, C-HF, and AER provided suggestions and revised the manuscript. All authors approved the manuscript and the version to be published.

## Funding

JN-B is grateful for the financial support of the Junta de Andalucía (Grant Number PAIDI-DOCTOR: DOC_00906). C-HF wishes to acknowledge the financial support of her research work under JSPS Grant-in-Aid for Early-Career Scientists (20K15477), Leading Initiative for Excellent Young Researchers from MEXT (2020L0277), FY 2021 President's Discretionary Grants, and SPDR Program. AER is grateful for the financial support of the National funds through FCT – Foundation for Science and Technology – under the Project UIDB/05183/2020.

## Conflict of Interest

The authors declare that the research was conducted in the absence of any commercial or financial relationships that could be construed as a potential conflict of interest.

## Publisher's Note

All claims expressed in this article are solely those of the authors and do not necessarily represent those of their affiliated organizations, or those of the publisher, the editors and the reviewers. Any product that may be evaluated in this article, or claim that may be made by its manufacturer, is not guaranteed or endorsed by the publisher.

